# Nonconsensual Neurocorrectives and Bodily Integrity: a Reply to Shaw and Barn

**DOI:** 10.1007/s12152-016-9275-6

**Published:** 2016-08-24

**Authors:** Thomas Douglas

**Affiliations:** 1 Oxford Uehiro Centre for Practical Ethics, Faculty of Philosophy, University of Oxford, Oxford, UK; 2 Brasenose College, Oxford, UK

**Keywords:** Neurocorrectives, Neurointerventions, Criminal justice, Criminal rehabilitation, Neuroscience, Chemical castration

## Abstract

In this issue, Elizabeth Shaw and Gulzaar Barn offer a number of replies to my arguments in ‘Criminal Rehabilitation Through Medical Intervention: Moral Liability and the Right to Bodily Integrity’, *Journal of Ethics* (2014). In this article I respond to some of their criticisms.

Brain-active drugs and other interventions that exert a direct chemical or physical influence on the brain are sometimes imposed by criminal justice systems, on criminal offenders, for the purposes of facilitating offender rehabilitation. I call such interventions *neurocorrectives*.

In existing ethical debate on neurocorrectives, it has often been assumed that they could only permissibly be used with the consent of the offender. In my article ‘Criminal Rehabilitation Through Medical Intervention: Moral Liability and the Right to Bodily Integrity’ I challenged this view [1].^1^ Perhaps the most obvious reason for thinking that nonconsensual neurocorrectives are impermissible is that they violate the offender’s right to bodily integrity. I sought to undermine this thought by drawing an ethical comparison between the interference with bodily integrity involved in nonconsensual neurocorrectives, and the interference with freedom of movement and association involved in incarceration. I suggested that, on the assumption that incarceration is permissible despite the severe constraints on movement and association that it involves, it is doubtful that neurocorrectives are *im*permissible in virtue of the interference with bodily integrity that *they* involve.

In fact, my assumption that incarceration is permissible was restricted to a particular, hypothetical kind of incarceration, which I called ‘minimal incarceration’. Minimal incarceration would consist in holding offenders

in institutions that placed serious and constant constraints on free movement and association, but otherwise exposed offenders to no greater risks to their health and security than average members of the unincarcerated citizenry, and took all reasonable steps to safeguard opportunities for political participation, legal representation and education (p. 105).

I assumed that it is sometimes permissible to subject criminal offenders to minimal incarceration, notwithstanding the interference with freedom of movement and association that this involves. It is very doubtful, however, whether it would be permissible to subject comparable *nonoffenders* to minimal incarceration. This suggests that committing certain types of crimes renders one morally liable to minimal incarceration, for instance, by weakening, waiving or activating an exception clause in one’s rights to freedom of movement and association. But if this is so, we might wonder whether committing those same crimes could also render one morally liable to the forms of bodily interference involved in nonconsensual neurocorrectives, for instance, by weakening, waiving or activating an exception clause in one’s rights to bodily integrity.

The challenge for the opponent of neurocorrectives is to explain why criminal offending deprives rights to freedom of movement and association of their normal protective force, but does not similarly deprive the right to bodily integrity of *its* protective force. I argued that the three most promising attempts to meet this challenge fail. I thus tentatively concluded that, *if* minimal incarceration is permissible, then nonconsensual neurocorretives are not impermissible in virtue of the kind of bodily interference that they involve.

Two papers in this issue raise a number of objections to my argument, and I am grateful to their authors—Elizabeth Shaw and Gulzaar Barn—for subjecting it to critical scrutiny [2, 3]. In this article, I respond to some of their objections, beginning with those offered by Shaw.

Shaw makes many careful and incisive criticisms. I do not have space to respond to all of them here, and in any case I do not have a response to all of them. However, I would like to raise some problems with what I take to be her four most powerful critiques.

## Shaw on The Epidemic Case

Much of my paper was spent assessing the *robustness claim*:

It takes more serious criminal offending for the rights to bodily integrity that protect against injection [the method via which I assumed neurocorrectives would be administered] to lose their protective force than for the rights to free movement and association that protect against minimal incarceration to lose theirs (p. 110).

If the robustness claim holds, then committing a crime could render one liable to the restrictions on free movement and association involved in minimal incarceration without also rendering one liable to the forms of bodily interference involved in nonconsensual neurocorrectives—namely, the injection of a drug. However, I argued that there is no good reason to think that the robustness claim holds. For instance, I suggested that appealing to first-order case-based moral intuitions does not clearly support it. I considered what Shaw calls the *epidemic case*:

*Jill* is infected with a novel strain of the Ebola virus, which could, if it spread, infect and kill many people. The only way to stop it spreading is to keep her in quarantine for three months, and, since Jill does not agree to this, the quarantine would have to be imposed against her will.

*Jane* is infected with a novel strain of the Ebola virus which could, if it spreads, kill many people. The only way to stop the virus spreading is to inject Jane with a drug. This drug will not cure Jane’s infection, but it will prevent the virus from infecting others. Jane does not agree to receive the injection, so it will need to be imposed against her will (p. 112, italics in original).

And I claimed that

it is not intuitively *clear* that the threshold for intervention should be higher in the case of Jane than in the case of Jill (pp. 112-13, italics in original)

where the ‘threshold for intervention’ was supposed to be the degree of harm that the virus would have to cause for the imposition of the intervention to be justified.^2^

I took this intuition to be significant since, if rights to bodily integrity were generally more robust than rights to freedom of movement and association, one might expect that a larger potential catastrophe would be required to justify infringement of the former than to justify infringement of the latter.

### Emergency and Non-Emergency Situations

Shaw raises two chief concerns regarding my appeal to this case. First, she notes that the case involves an emergency, in the sense that it involves a threat that is both grave and imminent. By contrast, she suggests, contexts in which we would consider imposing minimal incarceration or neurocorrectives are not emergency situations, since the offender poses a threat that is non-grave, non-imminent, or both. Thus, even if the rights to freedom of movement and association and bodily integrity at stake in the epidemic case are equally robust, this may imply little about the comparable rights at stake in criminal justice cases. It may be that

(i) the right to bodily integrity that protects against the injection of a drug is, in emergency situations, no more robust that one’s right to freedom of movement and association that protects against minimal incarceration

but that

(ii) the right to bodily integrity that protects against the injection of a drug is, in *non*-emergency situations, more robust that one’s right to freedom of movement and association that protects against minimal incarceration.

I agree that this is possible. However, I do not believe that it undermines the point I was attempting to make with the epidemic case, which was simply that intuitive reactions to that case do not clearly support the robustness claim—that the epidemic case does not serve as a counter-example to my rejection of the claim. If, as Shaw suggests, intuitions regarding emergency situations do not have any bearing on the robustness claim, which Shaw takes to refer to the rights that obtain in non-emergency situations, this will strengthen my point; it will suggest that appeals to the epidemic case never had any prospect of grounding persuasive objections to the claim.

Shaw’s criticism appears to be grounded on the thought that I was invoking the epidemic case in positive support for the robustness claim, whereas in fact it was playing a purely negative role. But it is interesting to consider whether it might play a positive role. Suppose one had the intuition about the epidemic case that the threshold for interference is precisely the same for Jill’s quarantine as it is for Jane’s forced treatment. Would this provide positive support for the robustness claim? Shaw appears to doubt this on the basis of the disanalogy that she has identified. But I am inclined to think that it would provide *some* positive support. Even though it is possible that (i) and (ii) both hold—i.e., that the relative robustness of the rights in question varies between emergency and non-emergency situations—there is at least some reason to suppose that they do not. There is, that is, some reason to suppose that conclusions about the relative robustness of these rights in emergency situations will carry over to non-emergency situations, and visa versa. This is because it is very unclear, theoretically, why the emergency/non-emergency distinction should make a moral difference.

It seems to me, then, that, notwithstanding possible moral differences between emergency and non-emergency situations, the epidemic case could provide some positive support for the robustness claim. However, I do not wish to claim that it does provide such support, because I do not myself have a clear intuition that the threshold for interference is the same for both quarantine and forced treatment in the epidemic case (though nor do I have a clear intuition that the thresholds are different). I thus prefer to stick to my initial, negative claim: that the epidemic case does not constitute a counterexample to my rejection the robustness claim.

At this point, Shaw might argue that there is a further problem: I have not considered the most promising type of counterexample to my view. Given the potential moral differences between emergency and non-emergency situations, I should have considered potential counter-examples that, like the relevant criminal justice cases, involve non-emergency situations.

This raises the question whether Shaw is right to think that criminal justice cases are *not* emergency situations. I am not sure that she is. Notice that in some cases where offenders are now incarcerated they do, or would if released, pose a grave and imminent threat to others. We might consider here cases in which a person in the late stages of planning a major terrorist attack or mass murder is identified and incarcerated, and will likely carry out the attack if released. Such a case might be quite closely analogous to my epidemic case.

Notice also that in some cases of slow-moving epidemics, decisions about quarantine and forced treatment do not involve an imminent threat (though the threat will almost always be a grave one). We could construct a variant of the epidemic case where the threat is grave but non-imminent. Such a case would be analogous, in terms of the gravity and imminence of the threat, to quite a few criminal justice cases, and I believe that my intuitive conclusions about the original epidemic case would hold for this variant case as well.

Nevertheless, I do agree with Shaw that my argument would have been strengthened by considering a broader range of potential counter-examples, including some that clearly involve non-emergency situations.

### Two-Option versus Three-Option Cases

Shaw’s second worry about the epidemic case is that it involves two separate comparisons between two options, rather than one comparison between three options. In the case of Jill, we are asked to compare quarantine (i.e., constraints on freedom of movement and association) with no quarantine, while in the case of Jane we are asked to compare the injection (an interference with bodily integrity) with no injection. Shaw rightly points out that in typical criminal justice cases there will instead be three options: constraints on freedom of movement and association (in this case via incarceration), interference with bodily integrity (for example, via injection of a neurocorrective), or neither. She fears that this disanalogy might prevent my conclusions about the epidemic case from carrying over to the criminal justice cases of interest.

Again, I agree that the disanalogy Shaw identifies is potentially morally significant. It could be that conclusions about the robustness of the relevant rights in two-option cases do not carry over to three-option cases. But I am inclined to respond to this precisely as I responded to the emergency/non-emergency disanalogy. First, if Shaw is right that one cannot make inferences about three-option cases from two-option cases, this will merely strengthen my claim that the epidemic case does not constitute a persuasive objection to the robustness claim. And, second, there is in fact some reason to believe that conclusions regarding two-option cases will carry over to three-option cases, meaning that there remains a possibility that the epidemic case could provide positive support for the robustness claim (though I would not wish to commit myself to this view).

Again, Shaw might argue that, if I wanted to consider the strongest potential objections to the robustness claim, I should have considered potential counterexamples that are as closely analogous as possible to actual criminal justice scenarios in which the use of neurocorrectives might be at issue. Thus, I should have considered three-option cases rather than two-option ones.

I believe, however, that there is in fact good reason to prefer two-option examples when assessing the robustness claim; three-option cases introduce a complication that we would do well to exclude, namely, they invite the thought that we could impose a disjunctive requirement on the offender—a requirement to accept *either* restrictions on free movement and association *or* bodily interference. Consider a case in which either quarantining an individual or subjecting him to treatment would avert an infectious disease catastrophe. The authorities thus face a choice in which the alternatives include imposing quarantine, imposing treatment, and imposing neither. Even if not stipulated as a further option (indeed, even if explicitly excluded), we might be tempted to think, regarding such a case, that the state could also entertain a fourth option: require that the infected individual *either* undergo quarantine, *or* receive treatment, with the choice left to that individual. Moreover, it might generally seem that imposing this disjunctive requirement would be preferable to imposing either quarantine or treatment without the individual being given any choice, since the disjunctive requirement leaves the individual with greater personal autonomy. Suppose that our intuition about this case was that it would never be permissible to impose quarantine or treatment without giving any choice, but it would, above some threshold level of harm averted, be permissible to impose the disjunctive requirement: quarantine or treatment. What would this show about the relative robustness of the right to free movement and association, and the right to bodily integrity? Would it suggest that these rights are equally robust? Not necessarily. It could be, for example, that the following hold: (i) the right to freedom of movement and association is less robust, so that it would, absent the possibility of imposing a disjunctive requirement, take a lesser catastrophe to justify impositions on freedom of movement and association than to justify interference with bodily integrity, but (ii) it is always preferable to impose the disjunctive requirement to imposing either quarantine or treatment alone, so that once the threshold for imposing quarantine is met, we should offer the alternative of treatment as well. Given this sort of possibility, it is difficult to draw conclusions about the robustness claim from three-option cases.

## Shaw’s Examples

Having argued I should have considered three-option cases that involve non-emergencies, Shaw then offers two such cases, and suggests these cases *do* tell against the robustness claim. The first of these cases is her *discipline scenario*:

The parents of Anne, who is 11 years old, discover that she is engaging in highly risky behaviour. Assume that there are two forms of discipline that are likely to prevent Anne from continuing to take these risks – grounding her or administering corporal punishment - and that they will probably be equally effective. Which option is preferable?

1. Allow Anne to engage in highly risky behaviour without any interference.

2. Ground Anne (i.e. interfere with free movement).

3. Administer corporal punishment to Anne (i.e. interfere with bodily integrity). (Shaw 2016, this issue)

Shaw suggests that “grounding is the best option, corporal punishment the next best, and doing nothing the worst option” (Shaw 2016, this issue). I agree. I also intuit that the threshold for grounding, in terms of the level of risk required to justify it, is lower than for corporal punishment. However, I do not think this counts against the robustness claim.

An initial difficulty is that the kind of bodily interference involved in this case is different from the kind of bodily interference that I was interested in and to which the robustness claim refers: the injection of a drug. I argued that the rights to bodily integrity that protect against different kinds of bodily interference may differ in their robustness. If this is correct, it may be that the that the right to bodily integrity at stake in corporal punishment *is* more robust than the right to free movement and association involved in grounding (or, for that matter, incarceration), but that the right to bodily integrity that protects against neurointerventions (viz., against the injection of a drug) is not similarly robust.

A further, and I think more serious, problem is that there are many ways of accommodating the intuition that the threshold for the permissible imposition of grounding is lower than the threshold for the permissible use of corporal punishment that do not require us to accept the robustness claim, because they do not invoke a right to bodily integrity. Shaw concedes herself that the threshold for corporal punishment may be higher because corporal punishment is more distressing, or (we may assume) less effective than grounding. I would add that it may be higher because it involves the intentional infliction of pain, which neither grounding, nor the injection of a drug (the kind of bodily interference that I was interested in) necessarily does.

Shaw goes on to provide the *driving scenario*:

Joe has a medical condition that causes him unpredictably to lose consciousness while driving. He lives in an isolated cottage in a remote area and relies on his car to meet friends and participate in activities he enjoys (although he does have access to medical and emergency services and can get food delivered etc. without his car). Imagine that the authorities only have the following three options. Which option is preferable?

1. Allow Joe to put other road users at risk, without any interference.

2. Compel Joe to stop driving.

3. Forcibly inject Joe with a drug that does not treat his underlying illness, but does prevent him from losing consciousness for the duration of his car journeys. (Shaw 2016, this issue)

Shaw believes that it would be better to compel Joe to stop driving than to forcibly inject him with the drug. I agree, and I also believe that the threshold for justifying a driving prohibition is lower than that for justifying forced imposition of the drug. But again, I believe all of this can be explained without accepting the robustness claim.

In this case, I believe the problem lies not in Shaw’s analogue for the bodily interference involved in neurocorrectives, but in her analogue for the constraints on movement and association involved in minimal incarceration. Minimal incarceration clearly involves constraints that would, if imposed on an innocent person, violate that person’s right to freedom of movement and association. By contrast, it is not clear to me that compelling Joe to stop driving involves such a rights-violation, notwithstanding Joe’s reliance on the use of a car to move around and maintain his associations. The reason for this is that the right to freedom of movement and association does not entail a duty on others to provide all means necessary to move and associate as one would like, or even as most people can. To see this, suppose that Joe’s brother John lives in an equally isolated place and is equally reliant on a car for movement and association. John has no medical condition, however he is very poor and can only afford to buy cars that are not roadworthy and pose a serious danger to others. It seems doubtful that the state violates John’s right to freedom of movement and association by prohibiting him from driving such dangerous vehicles.

I admit that, in the case of Joe, there may be a stronger basis for saying that a prohibition on driving would constitute a violation of his right to freedom of movement than there is in the case of John. However, it seems to me that there is nevertheless considerable scope for doubt as to whether there is any such rights violation in this case, and this may reasonably colour our intuitions about the driving scenario. It may make prohibiting Joe from using a car seem substantially less problematic than subjecting him to the kinds of constraints on movement and association involved in minimal incarceration.

## Shaw on Harm and Threats to Agency

Besides an appeal to intuition, I considered two other ways in which my imagined opponent might seek to defend the robustness claim—the claim that it takes more serious criminal offending for the rights to bodily integrity that protect against injection to lose their protective force than for the rights to free movement and association that protect against minimal incarceration to lose theirs. The first strategy would be to appeal to harm; one could argue that the sort of bodily interference involved in nonconsensual neurcorrectives (by assumption, the injection of a drug) is, or is typically, more harmful than the kinds of restrictions on free movement and association involved in minimal incarceration, so we should expect the rights that protect against it to be more robust. The second, more Kantian, strategy would be to appeal to agency; one could argue that the relevant kinds of bodily interference are (typically) more threatening to agency than the relevant restrictions on free movement and association. I argued that neither strategy succeeds, but Shaw comes to the defence of both.

### Harm

Regarding the appeal to harm, Shaw cites a study which found that psychiatric inpatients tended to prefer compulsory seclusion or physical restraint to compulsory treatment [4]. She takes this to suggest that they regarded minimal incarceration-like constraints on free movement and association as less harmful than bodily intrusions of the sort involved in forced treatment.

It seems to me doubtful that this evidence supports the robustness claim. First, these patients may have regarded forced treatment as more harmful not because of the bodily intrusion that it entailed, but in virtue of the mental intrusion involved. In that case, this evidence would, if anything, support a robust right to *mental* integrity, not to bodily integrity. Second, the patients may have objected to forced treatment because they perceived it to have more side-effects than is now typical for the injection of a drug; it is important to note that the study cited by Shaw was conducted on Swiss psychiatric patients hospitalized in 1981, a time at which psychiatric medications were typically less safe that they are today. Third, seclusion may have been seen as involving less harm than compulsory treatment because, in the context of psychiatric hospitalization, compulsory seclusion arguably did not involve a dramatic *reduction* in freedom of movement and association, which was already highly constrained. It is not clear that the difference between psychiatric hospitalization and seclusion, in terms of constraints on bodily and mental integrity, is comparable to the difference between minimal incarceration and non-incarceration.

### Threats to Agency

Consider now the second strategy for defending the robustness claim—the appeal to ‘threats to agency’. I distinguished two different ways in which an intervention might threaten an individual’s agency (pp. 115–16). First, it might constitute a communicative threat to agency, by expressing a denial of, or disregard for, the recipient’s agency. Second, it might constitute a causal threat to agency, by (expectably) resulting in a reduction in the recipient’s agency or sense of agency. I argued that there is no good reason to suppose that interferences with bodily integrity of the sort involved in nonconsensual neurocorrectives would, or would typically, constitute a greater communicative or causal threat to agency than restrictions on freedom of movement and association of the sort involved in minimal incarceration.

In defending this view, I appealed to the thought that bodily interference of the relevant kind would express a thoroughgoing disregard for the agency of the recipient only if the perpetrator of the intervention actually had an attitude of thoroughgoing disregard for the agency of the recipient. Shaw challenges this suggestion, arguing that certain interventions ‘objectively’—or at least, independently of the contingent subjective states of the perpetrator—express disregard for agency, or, as she prefers to put it, express disrespect. She gives, as examples of interventions that express such disrespect, flogging, rape, and performing a surgical procedure on someone without their consent. Shaw thinks that bodily interference of the sort involved in nonconsensual neurocorrectives belongs in this category too, and suggests that it might be comparable, in terms of disrespect expressed, to mild non-public flogging.

I concede that the messages expressed by our actions may to some extent be independent of the contingent attitudes of the actor. However, I am nevertheless unsure whether we should place bodily interference of the sort involved in nonconsensual neurocorrectives in the category of neurointerventions which express such subjectivity-independent disrespect. At least some comparable forms of bodily interference—such as that involved in forcibly administering vaccinations as part of pandemic control—seem to express no significant disrespect, or at least, much less than moderate non-public flogging, which, it seems to me, is disrespectful primarily because it involves intentional infliction of pain.

Shaw suggests that there is in fact one respect in which nonconsensual neurocorrectives express greater disrespect than flogging:

Arguably, the forced injection with a mind-altering drug would even involve an additional kind of disrespect that is not present in the flogging example. The injection sends out the message that the offender’s moral motivation is so deficient that it needs to be directly re-engineered. In contrast, flogging engages the offender’s agency as it is – either by providing him with a prudential reason to refrain from reoffending, if it is administered as a deterrent, or by responding in a retributive way to his free choice to do wrong.

The first thing to note here is that, even if Shaw is correct to think that nonconsensual neurocorrectives would express this message, they would do so in virtue of the mental (rather than bodily) interference that they involve. If nonconsensual neurocorrectives express the view that the offender is morally deficient, they do so because of the way in which they intentionally alter the offender’s mental—and more specifically moral—states and processes, not in virtue of the fact that they involve injecting a drug into someone’s body. So Shaw’s objection here is not an objection to the argument that I presented in the paper under discussion here [1], which was concerned only with *bodily* integrity-based objections to nonconsensual neurocorrectives.

Second, it is not obvious that expressing the view that an offender’s moral motivations are deficient, or even *so deficient as to require direct re-engineering*, is always impermissible. Suppose the offender subjected to the neurocorrective has moral motivations that *are* so deficient. In that case reasons to be honest with the offender may require us to express precisely this message. Moreover, expressing the message may in some respects promote the offender’s wellbeing—for example, by helping to undermine moral self-deception or grandiosity—so we may also have altruistic reasons to express the message. Finally, insofar as nonconsensual neurocorrectives can be used as a substitute for incarceration, their use may enable criminal justice systems to avoid expressing, through incarceration, the message that offenders are so morally deficient as to require general exclusion from society—a message that may seem at least as disrespectful as the message that worries Shaw.

## A General Response to Shaw

To the above specific responses to Shaw’s criticisms, I would like to add a further, more general response—a response that, if it succeeds, succeeds in diffusing *all* of Shaw’s criticisms, or at least, all of those that take the problem with nonconsensual neurocorrectives to lie in their violation of a right to bodily integrity.

In my defence of nonconsensual neurocorrectives against objections from bodily integrity, I assumed that nonconsensual neurocorrectives would involve a substantial degree of physical invasion; I assumed that they would, like most actual instances of chemical castration, consist in the injection of a drug. But notice that it is possible that nonconsensual neurocorrectives could involve lesser degrees of physical invasion. Thus, suppose that transcranial direct current stimulation (tDCS) could be used as a neurocorrective, say by being applied in a way that tends to suppress extreme impulses towards violent aggression. tDCS involves administering a very small electrical current to the brain via electrodes placed on the scalp, and it is regarded as one of the most promising avenues for treating Parkinson’s disease, among other neurological and psychiatric conditions [5, 6]. Though there is perhaps a sense in which tDCS is physically invasive—the electrical current does, after all, ‘invade’ the brain—it arguably involves a form a physical invasion that falls outside of the scope of a right to bodily integrity, or at least infringes on only a very weak right to bodily integrity. The kind of physical invasion involved here seems similar to that to which I subject someone to if I wake her from sleeping by turning on a light. Just as tDCS results in electrical energy penetrating a person’s skull, turning on a light results in electromagnetic radiation penetrating her skull—the light rays pass through the sleeping person’s eye lids, cornea and lens and reach her retina, which lies some distance within the skull. Yet we would hardly think that turning on a light to wake someone constitutes a (morally significant) infringement of the right to bodily integrity. (Of course, a tDCS-based nonconsensual neurocorrective might involve a morally significant infringement of *mental* integrity in a way that turning on a light does not, but that is not our question.)

This suggests that, even if Shaw is right that nonconsensual neurocorrectives that consist in the injection of a drug are ruled out as impermissible by the right to bodily integrity (something I should emphasise I still wish to deny), there may well be other nonconsensual neurocorrectives that are not so ruled out, because of the very minor form of bodily interference that they involve.

## Barn’s *Reductio*

Gulzaar Barn’s response to my paper contains a number of original and thought-provoking examples, and I am grateful to her for drawing these to my attention. It also contains a diverse range of objections, and again, I will not be able to respond to all of these here. I will focus on her chief criticism, which is that my argument constitutes a *reductio ad absurdum* of the permissibility of minimal incarceration.

As Barn notes, my argument relies on the assumption that the state may permissibly subject at least some criminal offenders to minimal incarceration, and that we might do so as a means to realizing the goal of rehabilitation, or whatever further goal rehabilitation serves (e.g. crime prevention, maintenance of security, or simply prevention of harm) (pp. 105–106). (Recall that minimal incarceration is a form of incarceration that involves serious and constant constraints on free movement and association, but otherwise exposes offenders to no greater risks to their health and security than are faced by typical members of the unincarcerated citizenry, and under which the state takes all reasonable steps to safeguard prisoners’ opportunities for political participation, legal representation and education (p. 105).)

Barn challenges my assumption that minimal incarceration is sometimes permissible. Much of her paper is devoted to establishing the ineffectiveness of minimal incarceration in realizing the goals that I attribute to it and to outlining its great moral costs. Further, she holds that if my argument succeeds, then the permissibility of minimal incarceration implies the permissibility of certain other interventions which seem intuitively abhorrent (call these the *unacceptable interventions*). This, she suggests, should lead us to reject the permissibility of minimal incarceration. She holds, then, not only that minimal incarceration is impermissible, but that my argument furthers the case against it by generating a *reduction ad absurdum* of its permissibility.

Let me begin with a point on which I agree with Barn: it is not clear or obvious or self-evident that minimal incarceration is sometimes permissible. I am, myself, unsure about its permissibility, since I agree with Barn that it has very great costs both for offenders and society. I do believe that its permissibility would be widely accepted (meaning that my argument should have dialectic force against many) and, independently, that it is plausible (meaning that its permissibility is worth entertaining as an ethical hypothesis), and it is for these reasons it seemed to me legitimate to make my assumption.^3^ However, the assumption is only an assumption, and if it turned out to be false this would not surprise or disappoint me. Nor would it undermine my conclusion, since that conclusion takes only a conditional form: *if* minimal incarceration is permissible as a means to realizing the goals that I specified, then nonconsensual neurocorrectives are not, by reason of the bodily interference that they involve, impermissible.

Here is another point on which Barn and I agree: if the permissibility of minimal incarceration indeed implied the acceptability of the unacceptable interventions, this would give us a strong and probably decisive reason to reject the permissibility of minimal incarceration. However, the permissibility of minimal incarceration does not imply the acceptability of the unacceptable interventions, or at least, my argument does not support such an implication. Barn thinks it does because she attributes to me what she calls the *parity claim*, according to which ‘if incarceration is permissible, then so too are other interventions that are no more harmful and no more threatening to agency’. I have two responses. First, I am not committed to the parity claim. And second, even if I were, I would not be committed to the moral permissibility of Barn’s unacceptable interventions.

Let me begin with the second of these responses. Barn spells out two of her unacceptable interventions. One of these—let’s call it *virtual punishment*—is described as follows:

Suppose that a new type of punishment was invented. Here, punishment is simulated as lasting longer, and a criminal’s own crime is simulated as being committed against them, in order to induce empathetic responses, which are said to facilitate rehabilitation (Barn 2016, this issue).

The second unacceptable intervention, which, following Barn, I will refer to as *medical curfew*, involves administering a sleep inducing drug:

let us imagine a medical corrective is invented that regulates a released criminal’s sleeping patterns, causing them to sleep for 12 hours of the day.. . This is implemented with a view to reducing their involvement in the night-time economy, which for them, previously involved drugs, gangs, and violence. With the help of this drug, the criminal will now arise, like clockwork, at 6am, and experience 12 hours of the day, until around 5pm where they will start getting tired and prepare themselves for sleep, and eventually fall fast asleep at 6pm. This ensures they can no longer be led astray by the night-time culture that they once used to occupy (Barn 2016, this issue).

These are ingenious cases, but I think it is doubtful that the parity claim conjoined with the permissibility of minimal incarceration implies the permissibility of either of the interventions that they describe. In both cases, it seems plausible that these interventions are more threating to agency than minimal incarceration. This is because both involve intentionally interfering with the minds of offenders in a way that minimal incarceration arguably does not.

Barn may argue that, if I take this line, then I must allow, contrary to what I claimed, that nonconsensual neurocorrectives are also more threatening to agency than minimal incarceration, because they too involve intentional mental interference. However, I do not think I must allow this. There are features of virtual punishment and medical curfew which lead me to think that these involve especially problematic forms of mental interference—forms that nonconsensual neurocorrectives certainly need not involve. For instance, virtual punishment presumably involves the intentional infliction of pain or suffering (this, I take it, is why Barn refers to it as a punishment), whilst medical curfew involves agential capacitation—it prevents the offender from exercising his agency. Nonconsensual neurocorrectives need involve neither.

Moreover, even if nonconsensual neurocorrectives are more threatening to agency than minimal incarceration in virtue of the kind of mental interference that they involve, this is perfectly compatible with my argument, which concerned only bodily integrity. I claimed only that nonconsensual neurocorrectives are not more harmful or more threatening to agency than incarceration *in virtue of the kind of bodily interference that they involve* (viz. the injection of a safe drug) and I used this to support the conclusion that, if minimal incarceration is permissible, then medical correctives are not impermissible *in virtue of violating a right to bodily integrity*. These claims are consistent with medical correctives being more threatening to agency or harmful in virtue of their mental effects and with their being impermissible for this reason, and indeed I explicitly allude to this possibility at the end of the paper.

Thus, even if I were committed to the parity claim, I would not be committed to the permissibility of Barn’s unacceptable interventions. In fact, however, I am not committed to the parity claim. Indeed, it is unclear why Barn attributes it to me, since I do not explicitly defend it. Perhaps she thinks it is the only way I can reason from the claim that

1. Interference with bodily integrity of the sort involved in nonconsensual neurocorrectives (viz. the injection of a drug) is not necessarily or even typically more harmful or more threatening to agency than the interference with freedom of movement and association involved in minimal incarceration

To my tentative conclusion that

(2) The right to bodily integrity of the sort at involved in the imposition of nonconsensual neurocorrectives is not more robust than the rights to freedom of movement and association at involved in the imposition of minimal incarceration.

Barn may take me to be implicitly appealing here to the view that

(3) Some right, *r*^*1*^, can be more robust than some other right *r*^*2*^ only if interferences of the sort ruled out by *r*^*1*^ are more harmful or more threatening to agency than interferences of the sort ruled out by *r*^*2*^*.*

Claim (3) brings us fairly close to the parity claim. It may imply that, if minimal incarceration is permissible despite the restrictions on freedom of movement and association that it involves, there can be no decisive rights-based objection to any interference *i* that is no more harmful or threatening to agency than minimal incarceration. This is because any rights that protect against *i* would, in that case, be no more robust than the rights to free movement and association involved in minimal incarceration, and those rights are, by assumption, insufficiently robust to create a decisive objection to such incarceration. Thus, there will arguably be no decisive rights-based objection to *i*, and if we add the assumption that there is no decisive non-rights-based objection to *i*, then we are forced to conclude that *i* must be permissible.

I do not defend or rely on (3), however. (3) holds that only differences in harmfulness or threat to agency can justify differences in the robustness of rights, but I hold only that such differences provide two possible ways of justifying such a difference. I allow that there may also be other ways of justifying this difference,^4^ and indeed I explicitly consider one in my article: one might appeal to intuitions regarding the moral permissibility of the kinds of interferences against which the rights protect. For reasons I gave in my article (pp. 111–13), I do not believe that an appeal to intuition can succeed in explaining why rights to bodily integrity of the sort at stake in nonconsensual neurocorrectives are more robust than rights to freedom of movement of the sort at stake in minimal incarceration. But it is perfectly possible that such an appeal might succeed in showing that some other rights at stake in, for instance, Barn’s unacceptable interventions, are more robust.

## Further Brief Responses to Barn

The *reductio* described above is the core of Barn’s argument, but she also makes a number of ancillary criticisms, and in this final section, I would like to respond briefly to five of these.

The first criticism concerns the orientation of my article towards the *consent requirement*, according to which neurocorrectives may permissibly be imposed on offenders only with their valid consent. I attributed this requirement to a number of other authors, and presented my own argument as an argument against it. Moreover, my argument begins from the rejection of an analogous requirement in relation to minimal incarceration; as discussed above, I assume that minimal incarceration can sometimes permissibly be imposed even without the valid consent of the offender.

Barn worries that, in motivating my assumption that minimal incarceration is sometimes permissible, I give too much weight to the rejection of a consent requirement in relation to such incarceration:

The reason that we incarcerate criminals. .. isn’t because we think it permissible to do anything to them without their consent, or because the Consent Requirement simply no longer holds for them in light of their offending. Rather, it is because of the particular aim of punishment that incarceration is intended to serve, be that rehabilitative, retributive or deterrent. This aim, whatever it may be, is what grounds the justifiability of incarceration. Consider; prisoners on America’s death row aren’t murdered because, or, for the reason that, their consent is violable. Rather, it is because of the intended retributive and deterrent effects (as unfounded as they may be) that this punishment is purportedly justified. That prisoners’ consent is violated in the process, is a corollary, or parallel effect, of the primary aims and justifications of the punishment. It is not the case that they become liable to punishment because of the violability of the Consent Requirement. Therefore, it does not seem enough to argue that the violability of their consent is what grounds another form of punishment (Barn 2016, this issue).

I agree with all of this and did not endorse the view that minimal incarceration is permissible *only because* there is no requirement to obtain valid consent before imposing it. Obviously, as Barn suggests here, a consent requirement is just one possible constraint on the permissibility of minimal incarceration. Its absence will not demonstrate the permissibility of such incarceration unless there is something to be said in its favour. This is where the goals of incarceration come in. It is precisely because the goals of incarceration are relevant to its justification that I took care to clarify my assumptions regarding those goals (p. 106).

Barn’s second ancillary criticism focusses on just those assumptions. As noted above, I assumed that both minimal incarceration and nonconsensual neurocorrectives would be administered for the purposes of facilitating offender rehabilitation, or whatever further goal rehabilitation serves (these further goals might include crime prevention, maintenance of security, or simply prevention of harm). Barn worries that this appeal to the ‘further goals’ of rehabilitation involves excessively broadening the concept of rehabilitation:

Douglas argues that two other goals of incarceration, incapacitation and deterrence, are also “commonly thought to serve the same higher objective as rehabilitation: namely, the *prevention of crime* or, more generally, the *maintenance of security*.” [Barn’s emphasis] This seems to problematically broaden the idea of ‘rehabilitation,’ however, and leads to the unpalatable conclusion that almost anything that fulfils these expansive goals can be done under the banner of rehabilitation (Barn 2016, this issue).

But there is no broadening of the concept of rehabilitation here. Rehabilitation remains a term for *one way* in which we seek to prevent crime or maintain security. I do not ‘define’ rehabilitation as crime prevention, as Barn later states, indeed I keep the them clearly distinct; I simply recognize that rehabilitation may be sought only as a means to the further goal of crime prevention.

Barn’s third ancillary criticism targets my assumption, for the sake of argument, that we have at our disposal neurointerventions that are effective as aids to offender rehabilitation. Barn objects:

It is no good to assume for the sake of argument that such drugs will aid rehabilitation. Whether such drugs really *can* facilitate rehabilitation is inextricably and interdependently tied up with their permissibility.[Barn’s emphasis] Indeed, whether such drugs would actually aid rehabilitation, and so be permissible, depends on how rehabilitation itself is defined, and what it is theorised to consist in (Barn 2016, this issue).

I agree that the permissibility of neurocorrectives will depend on their effectiveness; if I did not, then I would not have made any assumption regarding their effectiveness. However, I do not see how this undermines the legitimacy of my assumption. The very purpose of making such assumptions is to hold some determinants of permissibility fixed so that we can examine others. As noted above, I wished in this paper to specifically examine bodily integrity-based objections to neurocorrectives, so it is natural that I should want to exclude effectiveness-based objections from the scope of my discussion.^5^

A fourth ancillary criticism is that I appear to succumb to a kind of neural determinism according to which criminality is wholly determined by brain states and not at all by social factors. Barn tentatively attributes this view to me on the basis that my argument requires that criminal behavior is under the influence of neural dispositions, and that I nowhere either (i) explicitly endorse a mixed view according to which social factors also influence criminal behavior, or (ii) discuss any such social factors.

In fact, I do mention one important social risk factor for criminal behaviour—incarceration, which I note can have a criminogenic effect—and the reason I do not mention more is simply that my paper is addressed to the controversial question whether we should use biological influences to prevent recidivism, not to the (I take it uncontroversial) proposition that we should use social influences to do so. Given this aim, social influences on crime are, though powerful, irrelevant to my argument.

What is my actual view on the role of neural and social factors in generating crime? I do not have a well-developed position and am in fact not sure that the distinction is a perspicuous one, since presumably the influence of social factors on our conduct is, like all influences on our minds, in some why mediated by the neural states on which our minds supervene (I am not a mind-body dualist). However, my undeveloped view is that both biological and social influences play an important and interacting role in influencing criminality.

Finally, Barn objects to what she sees as my conflation of morality with criminality:

The language used in the paper, such as the intention that the “post-rehabilitation offender will be a *morally better person,*” [Barn’s emphasis] conflates morality with criminality, and posits criminals as morally deficient, as a general class, in virtue of their offending (Barn 2016, this issue).

Here I need to explain the context of the passage which Barn quotes. This passage concerned the definition of rehabilitation. I was speculating that one (though not the only) way of defining rehabilitation is as a kind of moral improvement. On this conception of rehabilitation, it is by definition the case that a successful rehabilitation programme results in a morally better person. I did also later suppose that rehabilitation might be pursued as a means to the prevention of recidivism, and if we understand rehabilitation to consist in moral improvement, such an intention would be legitimate only if there is some sort of relationship—though possibly a rather loose one—between at least some forms of immorality and criminality. However, the existence of such a relationship is very plausible, and assuming such a relationship does not amount to a conflation of the two concepts. Nor does it imply that criminals are ‘morally deficient, as a general class, in virtue of their offending’. The suggestions that rehabilitation might consist in moral improvement and might be pursued in part for the purpose of crime prevention are perfectly consistent with the view that some offenders are morally normal individuals placed in crime-promoting social circumstances. With respect to those offenders, criminal rehabilitation could then be understood as a way of conferring a kind of moral enhancement on offenders in order to inoculate them against such crime-promoting social factors.

I should emphasise, however, that I am in fact not sure that we ought to understand rehabilitation such that it consists in moral improvement. This is why I held open also the possibility that we ought to understand it more ‘thinly’ as simply becoming less likely to re-offend. It is also why I eschewed the terms ‘moral improvement’ and ‘moral enhancement’ as general labels for the kinds of interventions that I wished to examine.

## Footnotes

1 Unless otherwise specified, all page numbers refer to this article.

2 Shaw interprets me as making a claim about the relative strength of the rights at stake in these cases, and rightly worries that one cannot straightforwardly infer anything about the robustness of a right from its strength. One cannot infer from the fact that both rights (to bodily integrity, and to freedom of movement and association) can be overridden in these cases, that the threshold for overriding them (i.e. the size of the averted-catastrophe that is required) is the same. I agree. But Shaw’s worry here is based on a misunderstanding; my intuitive conclusion about these cases was not about whether these rights can be overridden, and thus was not about their strength, but was precisely about where the threshold for overriding them lies. I suggested that “it is not intuitively clear that the *threshold for intervention should be higher* in the case of Jane than in the case of Jill” (pp. 112–13, this emphasis not in original).

3 Barn speculates that I appeal to the widespread acceptance of the permissibility of minimal incarceration in motivating my assumption because I believe that its widespread acceptance supports its plausibility. This is not the case. The fact that a proposition is widely accepted can give us reasons to subject it to scrutiny and explore its implications even if it does nothing to increase its plausibility. For instance, I agree with Barn that (i) the moral permissibility of the death penalty is widely accepted, though (ii) it is not plausible. Nevertheless, I believe that the widespread acceptance of its permissibility could give us dialectic reasons to subject it to scrutiny and draw out its implications.

4 There may also be a difference in robustness though there is no way of justifying it.

5 Perhaps my assumption would be problematic if it could be shown that we never will have effective neurocorrectives, but Barn does not establish this.
